# Chronic abdominal pain after laparoscopic hernia repair due to mesh graft migration to the cecum: a case report

**DOI:** 10.1186/s13037-019-0220-6

**Published:** 2019-11-26

**Authors:** Abdullah AlShammari, Fatima Alyousef, Amal Alyousif, Zainab Alsulabi, Fatimah AlJishi, Isra Siraj, Hissah Alotaibi, Mohammad Aburahmah

**Affiliations:** 1 College of Medicine, Alfaisal University, P.O. Box 50927, Riyadh, 11533 Saudi Arabia; 2 Department of Surgery, King Faisal Specialist Hospital and Research Center (KFSH&RC), P. O. Box 3354, Riyadh, 11211 Saudi Arabia

**Keywords:** Hernia repair, Mesh graft migration, Cecum, Abdominal pain

## Abstract

**Background:**

Hernia repair with mesh graft is one of the most common procedures in general surgery. Mesh graft repair is the treatment of choice for umbilical and periumbilical hernias to minimize recurrence. One of the rare but serious complications is mesh graft migration to viscus. These complications can occur months to years after repair and their diagnosis can be challenging as they may present as vague abdominal pain only.

**Case presentation:**

A 74-year-old gentleman with multiple medical comorbidities was diagnosed with a para-umbilical hernia after which he underwent a laparoscopic hernia repair at our hospital using a mesh graft with no complications. On postoperative day 10, he presented to the emergency room (ER) complaining of colicky abdominal pain in the right iliac fossa for 1 day associated with diarrhea. A Computed Tomography (CT) scan of the abdomen and pelvis showed diffuse wall thickening of the cecum and terminal ileum with small free air worrisome for perforation. The decision was made in the ER to discharge him home on antibiotics. The patient then returned back multiple times to the ER for the same complaint along with bleeding per rectum for which he underwent further investigations. Months later, the patient presented again with the same symptoms. A CT scan revealed recurrence of a periumbilical hernia and thickening of the medial wall of the cecum with mesh graft material. The patient was then taken to surgery and intra-operative findings revealed migration of almost 50% of the mesh graft size to the cecum and part of the mesh graft was eroding the distal part of ileum just proximal to the ileocecal junction. Adhesolysis and limited right hemicolectomy with ileocolic anastomosis was done. The patient had an uneventful recovery after revisions surgery without any perioperative complications. He was discharged home on postoperative readmission day 5 and followed up at 2 weeks and 3 months without any delayed complications or subjective complaints.

**Conclusion:**

It is important to consider mesh graft migration to viscus as a cause of persistent abdominal pain and bleeding per rectum irrespective of the time of presentation post hernia repair.

## Background

Mesh graft repair is the treatment of choice for umbilical and periumbilical hernias to minimize recurrence [1]. Early complications of hernia repair include wound infection and chronic pain. Late and more serious complications include mesh migration, erosion, sigmoid colon perforation, small bowel obstruction, enterocutaneous fistula and abscess formation [1–3].

These complications can occur months to years after the hernia repair [2]. However, they are rare and it can be difficult to reach the diagnosis [4]. Mesh graft migration could be primary when the mesh graft is not securely attached to the surrounding tissue migrates through areas of least resistance, or secondary mesh graft migration occurs as a result of foreign body reactions that forms granulation tissue and eventually erodes the mesh graft and causes migration through trans-anatomical planes [5, 6]. Colonoscopy is the modality of choice for diagnosing mesh migration to the colon, though ultrasound and CT scan can also be helpful [4]. The migrated mesh graft into the colon can be asymptomatic, mimicking colonic polyp under endoscopy and can even be invisible on radiological imaging making the diagnosis of mesh migration challenging. Mesh migration to colon mostly presents with lower abdominal pain and tenderness. Other presentations include weight loss, anorexia, abdominal mass, wound site pain with discharge, symptoms of bowel obstruction or asymptomatic presentation [3]. Most common location for mesh graft migration is the urinary bladder. Mesh graft migrating into hollow viscous is rarely seen [4]. Management of mesh graft migration is by reoperation to remove the migrated mesh graft. Partial bowel resection might be needed in cases of transmigration of mesh graft [7]. It is vital to ensure that the mesh graft is positioned correctly and sutured to the surrounding fascia to avoid mesh graft migration [8]. Here in we report a case of mesh graft migration to the colon post umbilical hernia repair in a 74-year-old male presenting with abdominal pain and bleeding per rectum.

## Case presentation

A 74-year-old male, known case of diabetes mellitus (DM), hypertension (HTN), bronchial asthma and hypothyroidism who was diagnosed with para-umbilical hernia. The defect size was 1 × 2 cm, after of which he underwent a laparoscopic hernia repair at our hospital using a 15 × 20 cm eTPES mesh Bard® Composix™ E/X Mesh (Warwick, RI) with no complications. The patient had uneventful admission period and was discharged home in good condition at day 2 post-op.

**Fig. 1:**
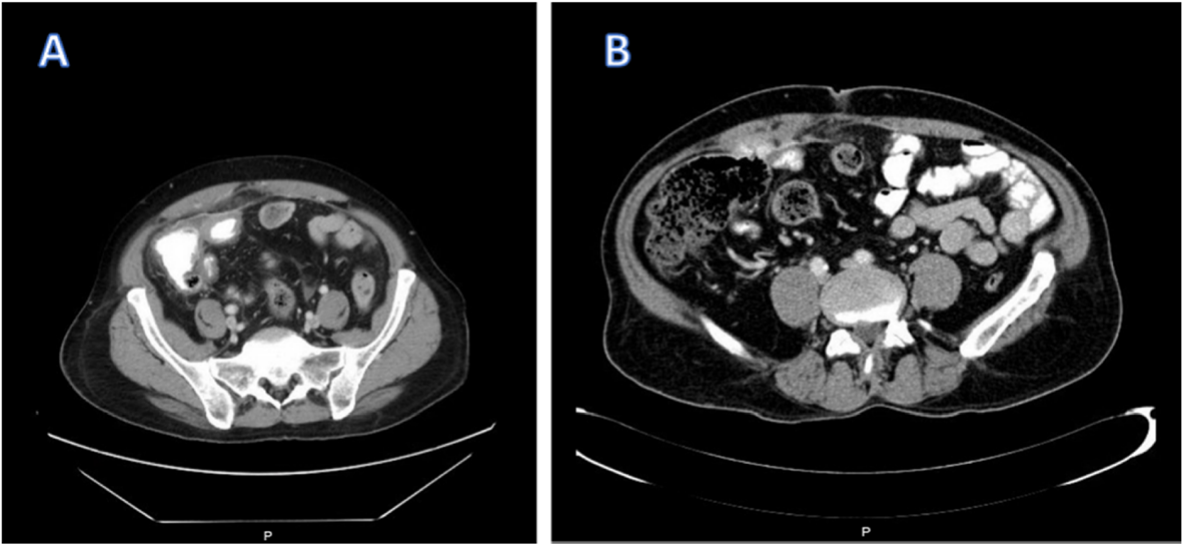
**a** There is a small free fluid area noted between the bowel loops with possible small localized fluid collection seen posterior to the mesh and anterior to the cecum. **b** Suspicion 1 mm focal defect within the anterior cecal wall

Postoperatively day 10, he presented to the Emergency Room (ER) complaining of colicky abdominal pain in the right iliac fossa for 1 day associated with diarrhea (5 times/day). No other symptoms were reported by the patient. On physical examination, patient had normal vital signs and generalized mild abdominal tenderness. The maximum tenderness point was at the right iliac fossa. A Computed Tomography (CT) scan of the abdomen and pelvis was done which showed diffuse wall thickening of the cecum and terminal ileum with abnormal configuration of the cecum and ill definition of its anterior wall with small free air worrisome for perforation (Fig. 1a & b). The decision was made in the ER to discharge him home on antibiotics.

**Fig. 2:**
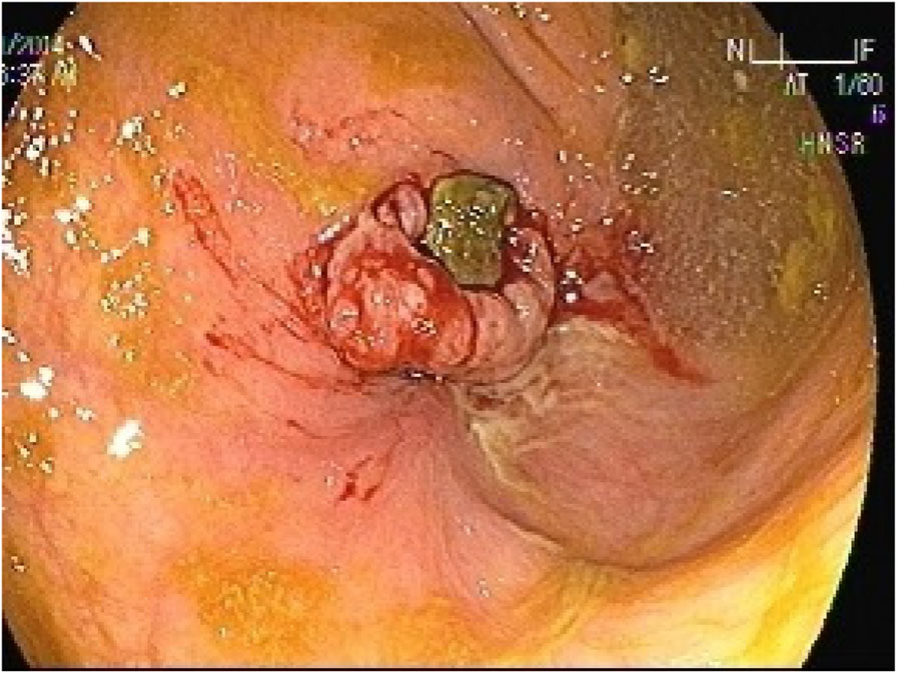
Colonoscopy picture showing small solid structure found in the cecum

Ten weeks post-operation, the patient presented again to his primary surgeon at our institute with the same complains where CT scan was ordered again and showed the same findings. Colonoscopy was ordered which showed small polyp that was removed and revealed benign adenoma, also showed a small solid structure in the cecum (Fig. 2). Biopsy of that solid structure showed granulating tissues.

**Fig. 3:**
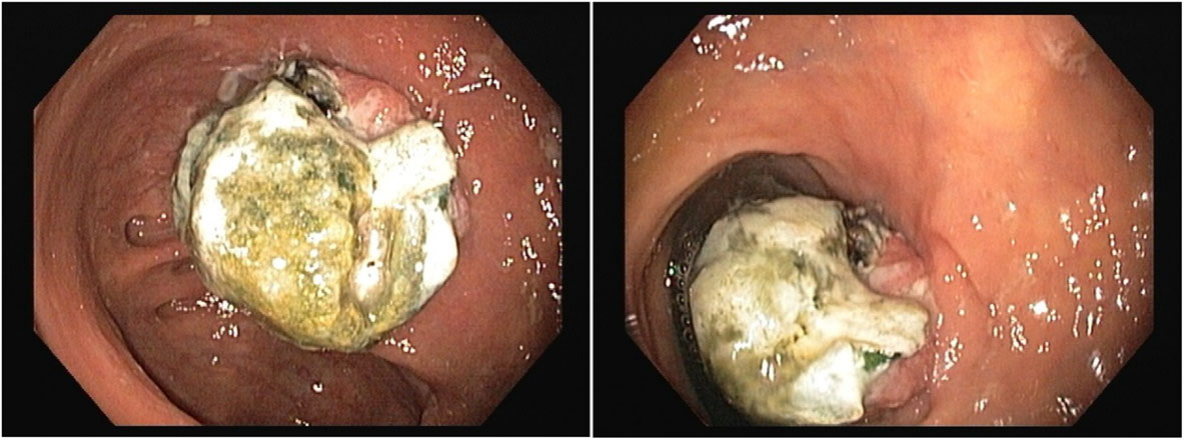
The colonoscopy picture showing polypoidal mass

Three months after surgery, the patient returned back to his primary surgeon complaining of bleeding per rectum for which he underwent further investigations including second colonoscopy. The colonoscopy showed a polypoidal mass that was biopsied and showed granulating tissues (Fig. 3). The patient was sent back to the surgeon’s clinic and reassured.

**Fig. 4:**
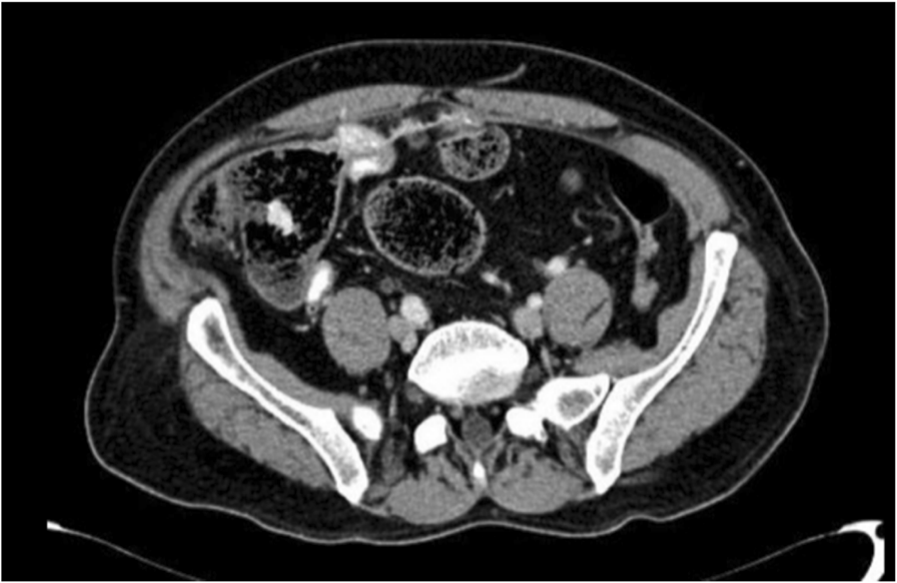
A mesh graft is noted at the level of the cecum and medial to that a dense opacity of about 7.7 × 6.7 × 4 cm noted, which is medially of low attenuation and contains some air bubbles. (Inflammatory process or infectious process at the level of the cecum medial to the mesh)

**Fig. 5:**
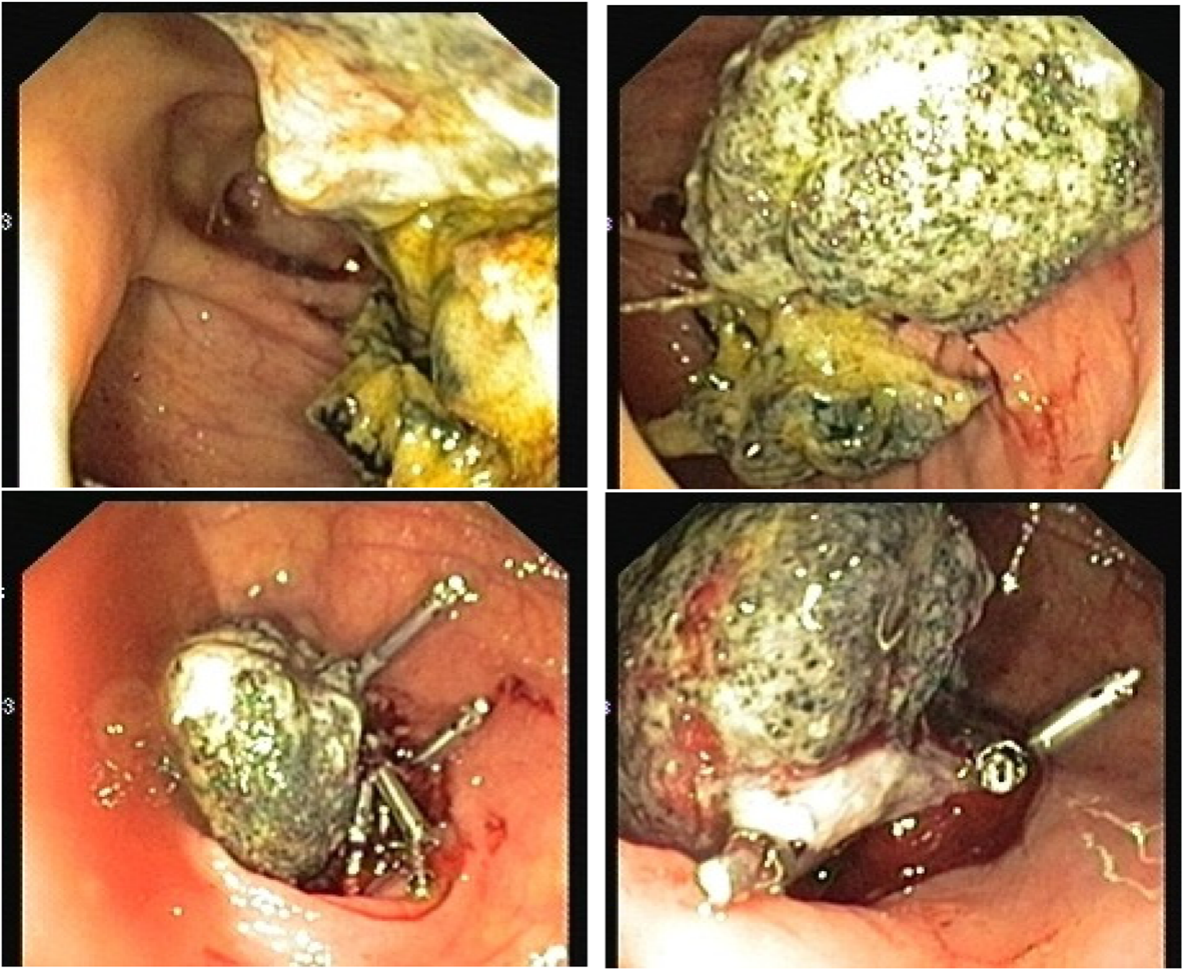
A colonoscopy showing a small mass in the cecum

Ten months post operation, the patient was seen in the outpatient clinic complaining of persistent vague abdominal pain and bleeding per rectum. CT scan of abdomen and pelvis was repeated which demonstrated opacity at the level of the cecum with dense opacity medial to it of low attenuation and contains some air bubbles (Fig. 4). Another colonoscopy was done which showed a small mass in the cecum (Fig. 5) that was biopsied and revealed to be granulating tissues, the patient was referred to the primary surgeon who reassured the patient and gave him a follow up appointment in one-year time.

**Fig. 6:**
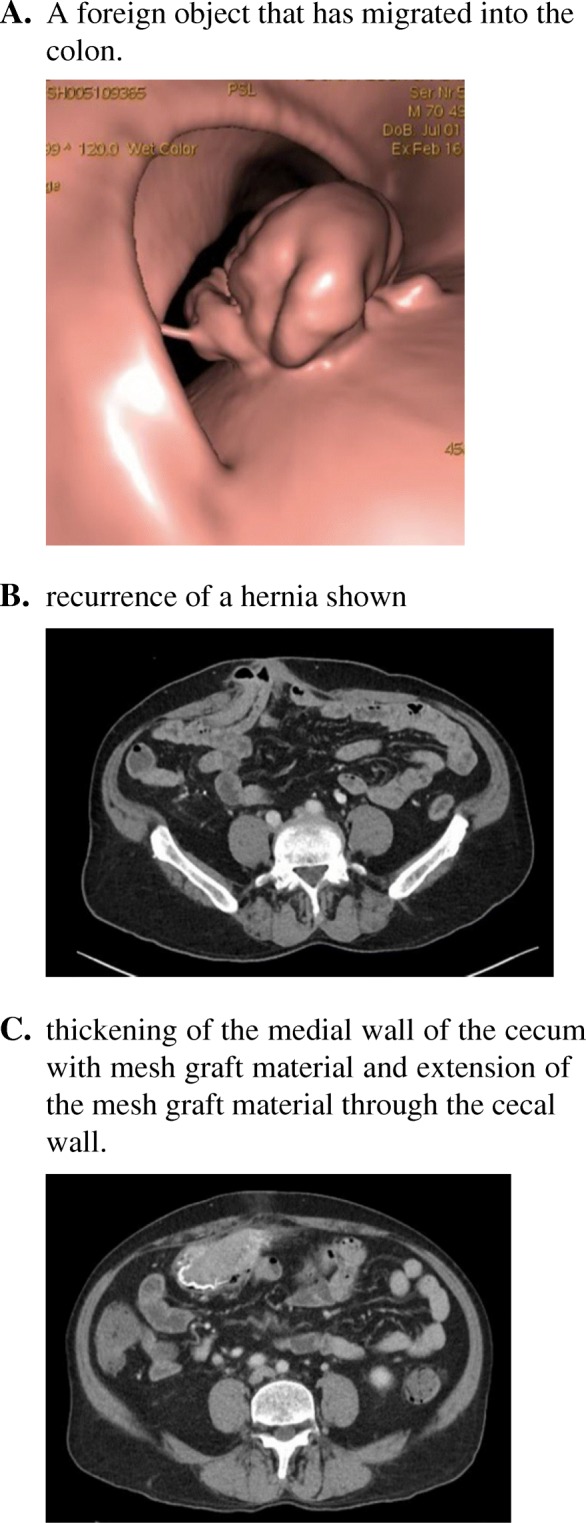
**a** A foreign object that has migrated into the colon. **b** recurrence of a hernia shown. **c** thickening of the medial wall of the cecum with mesh graft material and extension of the mesh graft material through the cecal wall

Follow up after more than 2 years of surgery indicated iron deficiency anemia and B12 deficiency. Thus, a colonoscopy was scheduled but the patient could not tolerate the bowel prep. A CT Colonography was ordered which showed a foreign object in the colon, possibly caused bleeding per rectum where the patient was referred back to the surgeon (Fig. 6a). A CT scan revealed recurrence of a periumbilical hernia and thickening of the medial wall of the cecum with mesh graft material that could suggest a complication of the previous ventral hernia repair with extension of the mesh graft through the cecal wall (Fig. 6b & c). Exploratory laparotomy was offered to the patient but he refused and was discharged against medical advice.

Ten months later, we received the patient in our service for the first time when he was complaining of persistent abdominal pain and bleeding per rectum. After reviewing his previous investigations, we told the patient about the mesh graft migration and we discussed with him the mandatory of exploratory laparotomy and mesh graft removal with bowel resection and the possibility of stoma creation at any point of surgery. The patient was informed that he would have higher rate of hernia recurrence and a mesh graft will not be used in this procedure to avoid further complications like wound/mesh infection due to the risk of bowel contents spillage in the surgical field on top of his other comorbidities like DM and bronchial asthma.

**Fig. 7:**
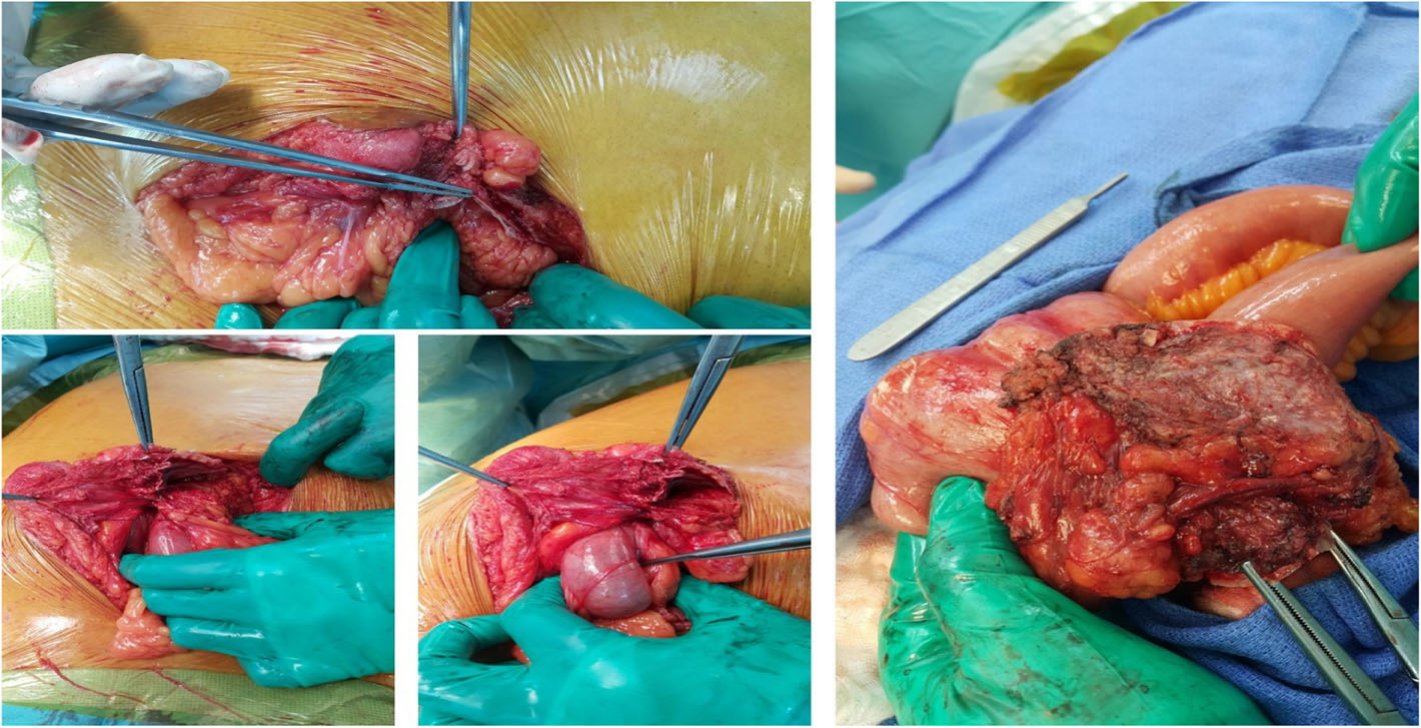
Intraoperative pictures during dissection of mesh adhesions to small bowel and colon

**Fig. 8:**
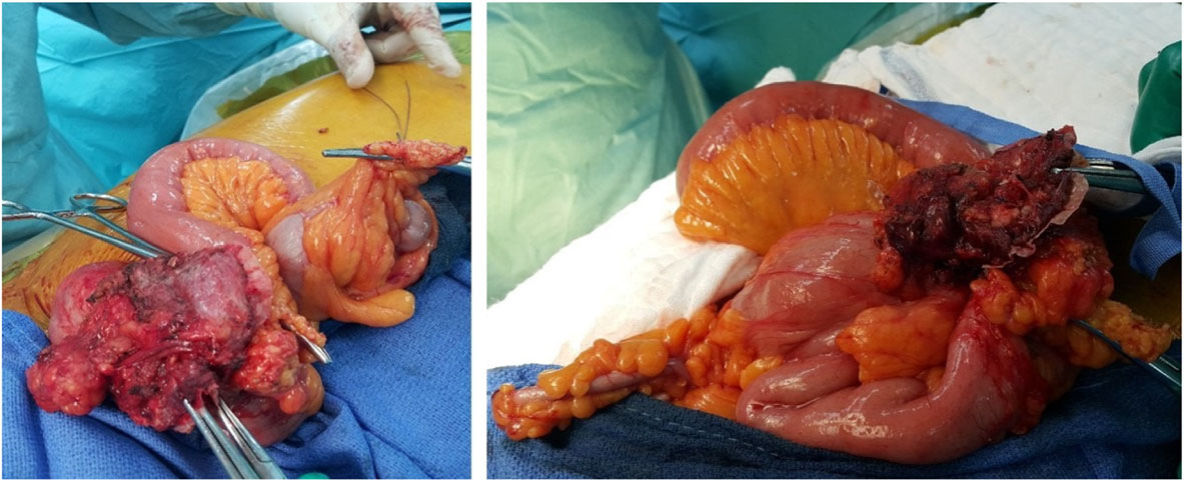
Small bowel and colon adhesion to mesh graft

**Fig. 9:**
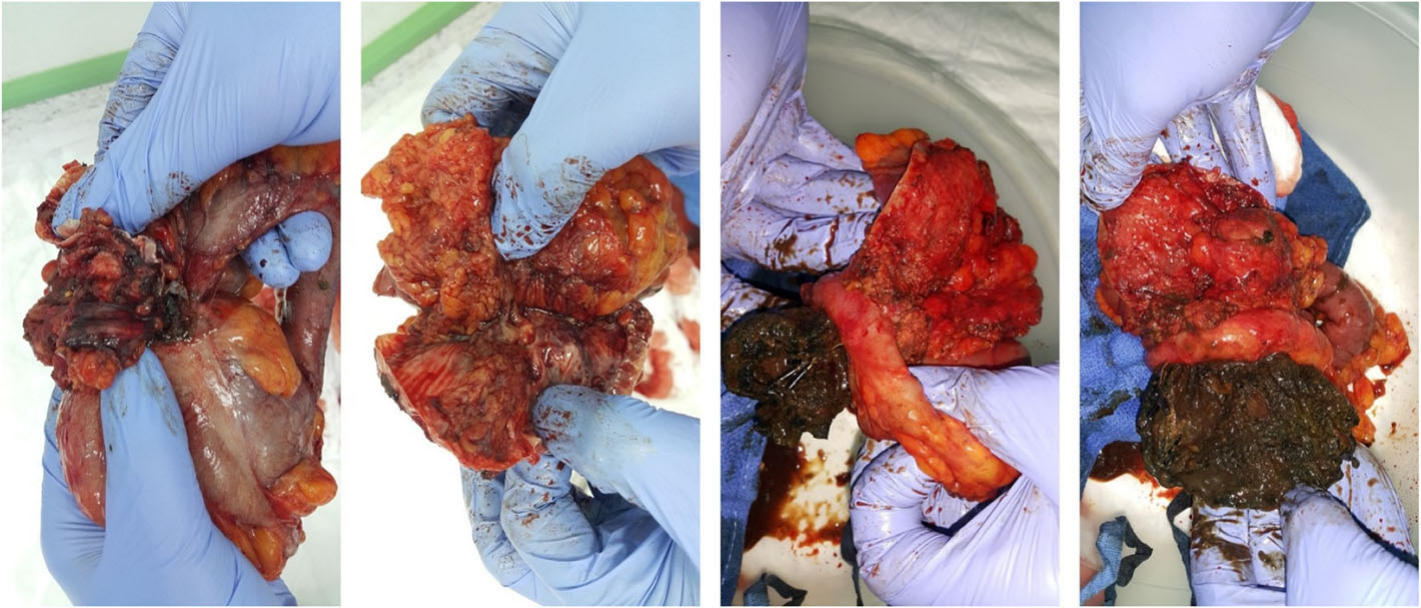
Specimen removed of distal ileum, cecum and proximal part of ascending colon with part of the mesh graft adhesed to the distal ileum bowel and the other part of the mesh graft migrated inside the cecum

The patient then was taken to surgery by his primary surgeon, and intra-operative findings revealed migration of almost 50% of the mesh graft size to the cecum and part of the mesh graft was eroding the distal part of ileum just proximal to the ileocecal junction. Adhesolysis and limited right hemicolectomy with ileocolic anastomosis was done. Fascia closure was obtained primarily without mesh graft application. Skin closure completed with skin clips (Figs. 7, 8 & 9). The patient had an uneventful recovery after revisions surgery without any perioperative complications. He was discharged home on postoperative readmission day 5 and followed up at 2 weeks and 3 months without any delayed complications or subjective complaints.

## Discussion

According to the literature, hernia repair with mesh graft is the gold standard technique for repairing umbilical and periumbilical hernias which helps in reducing the hernia recurrence rate [1]. Due to the nature of the mesh graft being a foreign object, the risk of complications increases after hernia repair such as hematoma, seroma, foreign body reaction, organ damage, infection, mesh graft rejection and fistula formation and rarely mesh graft migration [9]. The first reported case of mesh graft migration was by Herrera et al., in 1976, which migrated into the large bowel 2 years after the repair followed by the second case reported by Majeski et al., in 1998, that migrated and caused intestinal obstruction 30 years after the repair [1]. Still, the incidence of mesh graft complications has been reported up to 20%, where meshes migration being part of these complications and was recorded at an incident rate of 0.07% [10].

Complications can be classified as major complications which are life threatening that require emergency surgery and those that are clinically alarming complications which are significant but do not require an emergency set-up. Major complications include bowel obstruction, perforation and bleeding. Clinically significant complications include enterocutaneous fistula, sinus tract formation, intra-abdominal abscess, seroma and non-healing wound. Complications can also be classified in relation to hernia repair with definite relation or possible relation. Bowel obstruction is one such complication which can be possibly related to hernia repair if it is correlated with the patient’s prior abdominal surgery rather than the mesh graft itself. This can be confirmed by ruling out the presence of adhesion to the mesh graft [3, 9].

Similarly, most common long-term mesh graft related complications are bowel obstruction and sinus tract formation, keeping in mind that mesh graft size, which is determined by the size of the hernia, is a risk factor for the development of complications. Likewise, inflammation related to the mesh graft secures its repair due to fibrosis, compared to loose mesh without fibrosis leading to recurrence of the hernia. Therefore, Multiple measures to reduce the rate of inevitable mesh graft repair complications are considered during the repair [9, 10].

The risk factors and exact pathophysiology of mesh graft migration is still unknown. However, various factors such as inadequate fixation of the mesh graft to the fascia or sliding due to external forces and entry of the mesh graft into the abdomen from points of least resistance, or presence of adhesions and inflammations from previous surgeries can increase the risk of mesh graft migration. It is also hypothesized that the sharp edges of the mesh graft can induce an inflammatory reaction which can erode the parietal peritoneum and penetrate into the colon [7]. Mesh graft migration can occur acutely or over a period of years as a result of inflammation [1]. The surgical technique used, and the surgeon’s technical ability can increase the risk of infection and thereby increase the risk of fistula formation which can eventually cause mesh graft migration [4]. Additionally, the type of material used in mesh graft construction also determines the rate of infection and the tendency of the mesh graft to migrate [5]. Interaction of the mesh graft with the surrounding tissue varies depending on the property of the mesh resulting in different complications associated with different types of mesh graft. Mesh graft erosion and transmigration have occurred with different types of mesh graft including polypropylene plug mesh, polyester mesh, Polytetrafluoroethylene (PTFE) and dual layered PTFE and expanded polytetrafluoroethylene (ePTFE) mesh. Though, PTFE is associated with infections, polyester is associated with a chronic foreign body reaction and polypropylene is associated with an acute inflammatory response eventually resulting in fibrosis, further studies are needed to compare the incidence of mesh migration in different types of mesh graft [7].

On the other hand, in our experience explained above, one of the causing factors for such complication is using large mesh graft 20 × 15 cm for a small defect especially if it was not applied properly which allowed a gap between the mesh graft and the abdominal wall which caused entrapment of the bowel. Also, frequent visit of our patient to the emergency with worrisome CT finding make the diagnostic laparoscopy mandatory and that would save the patient from undergoing a major surgery like laparotomy and hemicolectomy.

Patients with mesh graft erosion and migration can present with acute intestinal obstruction, mass formation, bowel perforation and chronic abdominal pain between 1 and 20 years after repair [3]. The gold standard approach to diagnose mesh graft migration to the colon after hernia repair is colonoscopy [1]. While, ultrasound in the diagnosis of mesh graft migration is helpful, it is limited in most cases. CT, on the other hand, despite its better quality images is also limited in cases of infected mesh that can result in the formation of inflammatory granulation tissue [3]. In our case, the time to the development of mesh graft migration was 4 years where the patient presented to the ER several times with abdominal pain. The size of the mesh graft in respect to the size of the hernia in his primary surgery is of great concern to the development of his complications and should always be reflected. CT and colonoscopy were performed for him multiple times and eventually he was diagnosed with mesh graft migration to the cecum. Neglecting early CT scan findings despite their minor implications without proper interventions has added to the dilemma of early management to this patient. However, unlike most cases in the literature, there was no formation of fistula and our patient presented only with abdominal pain and bleeding per rectum. We believe that the cause of mesh graft migration was due to application of tacker that was prominent to attach to colon or long space between each tacker that allow colon entrapment. It might be prevented by applying trackers as standards and respecting the space between each tacker not to allow the colon to entrap or other abdominal structures and also not to forget an important factor which is using the right size of the mesh for the hernia size. Lastly, close follow-up to such patients should be of primary objective to achieve desirable outcomes. Our patient was initially managed conservatively for a long period of time with distant follow-up appointments and reassurances until the diagnosis of mesh graft migration was confirmed and the recurrence of periumbilical hernia occurred where he underwent a successful surgical repair.

## Conclusion

It is recommended for physicians to be mindful of the fact that imaging could be misleading towards the diagnosis of mesh graft migration making the diagnosis of mesh graft migration left unnoticed or overlooked. It is important to consider mesh graft migration as a cause of abdominal pain and bleeding per rectum irrespective of the time of presentation post hernia repair. Our case didn’t demonstrate a typical presentation like those reported in the literature with fistulas. This case is a reminder that mesh graft migration cases are not always associated with fistulas.

## Data Availability

Data sharing is not applicable to this article as no datasets were generated or analyzed during the current study.
